# Blood lead levels in children residing in a 115-year old urban settlement in Harare, Zimbabwe: a cross sectional study

**DOI:** 10.1186/s12887-023-03886-6

**Published:** 2023-02-10

**Authors:** Svitsai Chagonda, Itai James Blessing Chitungo, Cuthbert Musarurwa, Terence Nyamayaro

**Affiliations:** 1 Department of Laboratory Diagnostic and Investigative Sciences, Faculty of Medicine and Health Sciences, Chemical Pathology Unit, University of Zimbabwe, Harare, Zimbabwe; 2 Department of Biomedical Laboratory Sciences, School of Health Sciences, College of Medicine and Health Sciences, University of Rwanda, Kigali, Rwanda

**Keywords:** Blood lead, Children, Harare, Environmental, Exposure, Zimbabwe

## Abstract

**Background:**

Elemental lead (Pb) toxicity in children, irreversibly affects their growth and development. We assessed the prevalence of high blood Pb levels (BLL) in children living in a potentially high risk residential area and also assessed Pb levels in environmental specimens.

**Methods:**

This cross sectional study measured blood lead levels (BLL) in 86children living in Mbare, a densely populated suburb in Harare, Zimbabwe, characterised by dwellings progressively constructed from 1907 through to the 1940s, before the ban of leaded paint. Study participants of both genders were under 6 years of age. Potential risk factors of Pb poisoning were assessed. Pb levels were also assessed in soil, water and paint chip specimens from the study area.

**Results:**

The mean (standard deviation) BLL was 4.3 ± 0.75 g/dL. Twelve (13.95%) participants had BLL of > 5.0ug/dL. Our results showed no significant association between BLL and household income, participant behaviour/habits/activities, sources of drinking water, and the types of cookware used to prepare meals in their households.

**Conclusion:**

Mean BLL observed in the current study were higher compared to those of children of similar age groups in the United States, suggesting that Pb contamination may be more ubiquitous in the Mbare flats area, potentially predisposing these children to impaired development.

## Introduction

Toxicity due to elemental lead (Pb) exposure has been documented since as early as 370 BC, although it was only around 1 AD that Pb exposure was associated with adverse health outcomes [1]. Ancient Egyptian papyrus scrolls refer to possible toxic effects of Pb exposure [2]. Pb is ubiquitous and naturally occurring in trace amounts, but its extensive industrial applications have made it the most widely scattered toxic heavy metal. Historically, leaded petrol was the leading contributor to elevated BLL. Lead-based paints were also a major contributor to environmental Pb contamination until use of lead based paints was banned in 1978 [3]. A subsequent universal ban was imposed on the use of Pb in other products including, children’s toys and petroleum fuels [4]. However, despite this ban, occupational exposure and persistence of lead use in various products remain critical risk factors for elevated BLL [5]. Lead is a potent and progressive neurotoxin with high environmental persistence. Prolonged exposure to Pb at high levels can be fatal, but long-term low-level exposure in children is reportedly associated with irreversible developmental abnormalities [6, 7]. Children, along with pregnant women and their developing foetuses, are most susceptible to the effects of lead poisoning due to their high bone and tissue turnover [3, 8, 9].

Blood lead level is the established biomarker of Pb exposure. After the widespread ban of Pb use, progressive marked decreases in BLL have been reported in the USA and elsewhere from the 1970s' [10, 11]. The minimum acceptable cut-off point for BLL has transitioned from 60 µg/dL in the 1960s to 10 µg/dL in the 1990s and 5 µg/dL by 2015 for all age groups [5]. However, to date a safe BLL has not been clearly defined [12, 13]. The decline in population BLL is attributed to a combination of national regulations and public health awareness campaigns instituted to mitigate the effects of Pb exposure. Such measures have however, not been holistically implemented in many developing nations, including Zimbabwe. Risk factors predisposing children to Pb poisoning include among others; ethnicity, iron deficiency, fasting state and residing in older housing [14]. In Zimbabwe, buildings that were constructed prior to the international Pb ban, were painted using lead-based paints and remain inhabited to date. Inhabitants of such houses are therefore potentially exposed Pb-laden paint flakes. Overall, younger children are especially susceptible to elevated BLL due to their hand-mouth behaviour, exposure to contaminated soil, and pica behaviour. Furthermore, children absorb lead comparatively more efficiently from the gastrointestinal tract than adults (40% compared to 5% -15% for adults) and have an immature detoxification system which further confounds elevated BLL [12].

Elemental Pb persists in the environment mainly from existing products and infrastructure that predate the international ban and also in areas that were previously heavily contaminated with environmental Pb [3, 15]. The adverse consequences of Pb exposure, necessitate the identification of at-risk individuals and the need to adopt mitigatory preventive measures to minimise adverse outcomes. The study site for the current study is characterised by aged housing units constructed before the banning of lead in paint. Many poorly regulated cottage industries also operate from the study site environs. Additionally, portable water to this suburb is conveyed through pipes installed in the early twentieth century connected using Pb solder. Heavy vehicular traffic, and a nearby heavy industrial site further contribute to possible environmental Pb pollution in the study area. The present study investigated the prevalence of elevated BLL and associated risk factors of Pb toxicity in children living in Mbare apartments in Harare, Zimbabwe.

## Materials and methods

In this cross-sectional study, 86 apparently healthy children aged six years and below were randomly recruited from December 2020 to February 2021. The study was carried out in Mbare a high-density suburb in Harare, Zimbabwe. Mbare, one of the oldest suburbs in Harare was developed to house indigenous workers in the then newly established city of Salisbury, the capital city of then Rhodesia. The suburb consists of semi-detached houses and several apartment blocks originally meant for bachelor employees. Altogether there are 14 three storey apartment blocks, with each block consisting of 10–14 apartment units. Stratified random sampling was used to recruit study participants. Of the 14 apartment blocks, 5blocks were randomly selected and nine apartments were randomly chosen within each block as study sites. The first apartment was randomly selected, and subsequently, every seventh apartment was eligible for inclusion. In each apartment, two children were randomly selected, and if there were no children or if the children did not meet the enrolment criteria, the next apartment became eligible. All children under six years who had lived in the study site from birth were eligible for enrolment.

After obtaining written informed consent from their parents/guardians, assent was also sought from eligible children. A questionnaire eliciting clinicodemographic, socioeconomic, and the child’s play habits was administered. Each child’s weight and height was measured using standard calibrated tools. Subsequently, each eligible participant donated at most 3millilitres of whole blood obtained by venipuncture and placed into an ethylenediaminetetraacetic acid (EDTA) tube (Becton Dickinson, New Jersey, USA). Environmental samples that included scrapings of flaking paint (10grammes) from each participating apartment; and 20grammes soil samples (from identified participant play areas) were also collected. Water samples (100 ml per sample) that included puddle water, communal borehole water and communal municipal tap water were also collected. Two specimens each of- puddle water, drinking water, paint chips, and soil- were collected from each site.

### Laboratory analyses

All laboratory ware was washed with 20% nitric acid and rinsed in deionised water to remove any contaminating environmental Pb prior to use. Blood samples were stored at 2 – 8.^0^C and analysed within one week of collection. Thereafter, all specimens were stored for a maximum of 14 days and only disposed of by incineration after result verification. Both blood and environmental samples were analysed by flame atomic absorption spectrophotometry (AAS) with an extraction step prior to analysis (Varian, Model AA-1275 series, California, USA). Briefly, in AAS, the Pb in the sample absorbs incident light of specific wavelength from a hollow cathode lamp, and the intensity of the residual incident beam is measured at 283.3 nm [16, 17]. The attenuation of the incident beam intensity is proportional to the amount of Pb in the sample. The analytical sensitivity of the assay was 1 µg/dL. In all cases, the principles of good clinical laboratory practice were observed [18]

### Environmental sample preparation

Fifty millilitres of water samples from puddles, and drinking water taps were digested using hot reagent grade concentrated nitric acid before dilution with deionised water prior to analysis [19, 20]. Bioaccessible lead was extracted from soil and paint chip samples using the aqua regia method [21]. Briefly, 5 g soil or paint chips were added to a 50 mL acid-washed glass container and mixed with 37.5 ml of concentrated hydrochloric acid and 12.5 ml of 55% nitric acid. The mixture was left overnight, and the supernatant was subjected to analysis the following day.

### Blood specimens

For blood Pb analysis, 0.4 ml whole blood was mixed with 1.6 ml of 5% nitric acid in a polypropylene tube and shaken vigorously. The mixture was allowed to stand for 60 min before centrifugation at 2000 g for 15 min. Lead was analysed on the supernanatant which was stored at 2–8 °C if it could not be immediately analysed. The assay was standardised using a certified reference standard obtained from RICCA Chemical Company (Texas, USA).

### Data analysis

Data were analysed using STATA version 13.0. (StataCorp, College Station, Texas, USA). Categorical data were summarised by count and proportion whilst parametric continuous data was summarised using mean ± standard deviation (SD). The two-sample t-test was used to compare means of normally distributed data and the Fisher’s exact Chi-square was used to compare proportions. Logistic regression analysis was used to assess the association between the various study variables and the risk of a child having a blood lead level above 5 µg/dl. In all statistical comparisons, α was set at 0.05.

## Results

A total of 86 children were enrolled into the study. The clinicodemographic characteristics of the study participants (overall and by gender) are presented in Table 1.

**Table 1:** Clinicodemographic characteristics of the study participants

Variable	Overall	Male	Female	*p*-value
BLL µg/dL mean ± SD	4.3 ± 0.75	4.3 ± 0.78	4.4 ± 0.71	0.589
BLL > 5 µg/dL	12(14.0)	5(9.8)	7(20.0)	0.215
Gender	86	51 (59.3)	35 (40.7)	0.0899
Age (months) mean ± SD	37.3 ± 18.3	39.1 ± 15.4	34.5 ± 21.9	0.258
Weight (kg) mean ± SD	11.97 ± 3.52	12.4 ± 3.3	11.3 ± 3.8	0.152
Height (cm) mean ± SD	89.4 ± 13.3	91.1 ± 11.0	86.8 ± 15.8	0.142
Pica	34(39.5)	17(33.3)	17(48.6)	0.182
Soil eating	27(31.4)	13(25.5)	14(40.0)	0.166
Burning Rubbish Exposure	63(73.3)	35(68.6)	28(80.0)	0.323
No Hand washing	35(40.7)	17(33.3)	18(51.4)	0.119
**Toy preference**				
Dolls	23(26.7)	1(4.4)	22(95.6)	< 0.001
Plastic	37(43.1)	32(86.5)	5(13.5)	
Random	26(30.2)	18(69.2)	8(30.8)	

Key: All values n(%) unless otherwise stated

*SD* Standard deviation, *BLL* Blood lead levels, *n* Number, *cm* Centimetres, *kg* Kilograms

The overall mean ± SD blood lead level was 4.3 ± 0.7 µg/dL. There was no significant difference in mean ± SD BLL by gender; 4.3 ± 0.78 in males and 4.4 ± 0.71 in females, *p* = 0.589. Only 12 (14.0%) of the overall participants had BLL > 5 µg/dL. Of these, 7 (20.0%) were female, but there was no significant difference in the proportion of participants with BLL > 5 µg/dL by gender.

Although there were more male participants *n* = 51(59.3%) compared to females, the difference was not statistically significant (*p* = 0.08). The overall mean ± SD age of the study participants was 37.3 ± 18.3 months and there were no statistically significant differences in participant age by gender *p* = 0.258. Similarly, no significant differences were observed in any of the other parameters except in the proportion of toy preference where more girls preferred dolls to other toy types and a majority of boys preferred plastic and random objects, such as homemade steel wire toy cars, brick or discarded empty bottles, *p* < 0.001.

The participants were stratified by various putative risk factors for elevated BLLand mean BLL were compared between the various variables. The results are presented in Table 2.

**Table 2:** Comparison of BLL by putative risk factors

		**BLL µg/dL mean ± SD**		*p* value^*^
**Characteristic**	**Yes n (%)**	**Yes**	**No**	
History of unexplained abdominal pain	19(22.1)	4.6 ± 0.90	4.3 ± 0.69	0.106
Nursery school attendance	13(15.1)	4.4 ± 0.84	4.3 ± 0.74	0.842
Predominantly playing outside	49(57.0)	4.3 ± 0.83	4.4 ± 0.64	0.778
Sucking of fingers	11(12.8)	4.3 ± 0.90	4.3 ± 0.73	0.891
Pica Behavior	34(39.5)	4.4 ± 0.75	4.3 ± 0.76	0.771
Eating soil	27(31.4)	4.5 ± 0.74	4.3 ± 0.74	0.188
Exposure to batteries	7(8.1)	4.8 ± 1.05	4.3 ± 0.73	0.627
Playing around old cars	7(8.1)	4.1 ± 0.55	4.4 ± 0.76	0.383
Exposure to burning rubbish	63(73.3)	4.4 ± 0.75	4.2 ± 0.73	0.221
Exposure to petrol	4(4.7)	4.6 ± 0.66	4.3 ± 0.76	0.566
Washing hands after playing	51(59.3)	4.3 ± 0.78	4.5 ± 0.70	0.249
Parental occupational exposure	13(15.1)	4.5 ± 0.86	4.3 ± 0.73	0.550
Exposure to medicinal herbs/cosmetics	7(8.1)	4.2 ± 0.47	4.4 ± 0.77	0.508
Age ≤ 24 months	25(29.1)	4.3 ± 0.67	4.3 ± 0.75	0.985

Key:*BLL* Blood lead levels, *SD* Standard deviation. Differences in mean BLL were evaluated using a two-sample t-test with equal variances. *p*-value^*^ compares the mean BLL for the two groups

Although there were no significant differences in the mean ± SD BLL for any of the risk factors considered, BLL were marginally higher in children with a history of unexplained abdominal pain, those exposed to burning rubbish and in children who did not regularly wash their hands after coming from playing outside the house. However, the differences were not statistically significant in all instances *p* > 0.05.

Univariate logistic regression analysis was used to determine predictors of a high BLL (≥ 5.0 µg/dL) among the putative risk factors. Odds ratios were determined using univariate logistic regression analysis with BLL ≥ 5.0 µg/dL as the outcome variable and various putative risk factors as the explanatory variables. The results are presented in Table 3.

**Table 3:** Clinical and sociodemographic characteristics of study participants and association with BLL ≥ 5.0 µg/dl

Variable	n(%)	OR (95%CI)
**Monthly Household Income USD**		
< 20	59(68.6)	0.79 (0.3–2.02)
10–100	19(22.1)	
101–150	6(7.0)	
> 150	2(2.3)	
**Toy preference**		
Dolls	23(26.7)	1.57 (0.68–3.64)
Plastic	37(43.0)	
Random	26(30.2)	
**Positive history of unexplained abdominal illness**	19(22.1)	3.06 (0.84–11.1)
**Attending Nursery School**	13(15.1)	2.13(0.49–9.25)
**Predominantly play outdoors**	49(57.0)	1.07(0.31–3.67)
**Sucks fingers**	11(12.8)	1.45(0.27–7.68)
**Positive history of Pica**	34(39.5)	1.64(0.48–5.59)
**Regularly eats soil**	27(31.4)	2.52(0.73–8.72)
**Exposed to batteries**	7(8.1)	1.03(0.11–9.39)
**Exposed to burning rubbish**	63(73.3)	1.98(0.40–9.81)
**Exposed to petrol**	4((4.7)	2.15(0.21–22.6)
**Washes hands-on returning indoors**	51(59.3)	0.64(0.19–2.19)
**Parent/guardian occupational exposure to lead**	13(15.1)	1.15(0.22–5.95)

Key: *OR* Odds Ratio, *CI* Confidence Interval

None of the putative risk factors under study significantly predicted the risk of BLL > 5 µg/dL. However, although toy preference was not significantly associated with BLL > 5 µg/dL and there was no overall difference in BLL by toy preference (*p* = 0.805), mean ± SD BLL were significantly higher in children who played with random toys, 4.6 ± 0.79 compared to those that played with plastic toys 4.2 ± 0.70; *p* = 0.04 (data not shown).

Putative risk factors for BLL > 5 µg/dL that yielded *p* < 0.6 in univariate logistic regression analysis were fitted into multivariate logistic regression analysis models. After adjusting for toy preference and history of exposure to petrol, a history of unexplained abdominal pain was a significant predictor of BLL > 5 µg/dL OR4.39(95%CI: 1.07–17.9).

The results obtained for lead levels from environmental specimens are presented in Table 4.

**Table 4:** Pb level in environmental specimens

**Environmental Specimen** (10 Samples each)	**Pb level** **Mean ± SD**	**Range**	**WHO Recommended Levels**
Soil (mg/kg)	89.8 ± 71.1	3.34 – 177.5	250-400 mg/kg
Drinking water (community boreholes) (µg /L)	1.1 ± 0.7	0.3 – 2.0	Up to 10 µg /L
Drinking water community tap (µg /L)	1.9 ± 0.9	0.7 – 3.3	Up to 10 µg /L
Paint chips (mg/kg)	82.0 ± 57.4	9.64 – 169.2	Up to 600 mg/kg
Water from puddles (µg /L)	1.1 ± 0.5	0.6 – 1.8	

Key: *SD* standard deviation

The highest mean concentration of Pb in environmental specimens was detected in soil specimens at 89.8 mg/kg, while the lowest mean concentration (1.1 µg/L) was reported in both drinking water from community boreholes and from water puddles.

## Discussion

Our study is the first to interrogate BLL in Zimbabwean children. The study yielded some surprising findings. The study site should theoretically be heavily contaminated with environmental lead, yet only 12 (13.95%) of the 86 study participants had BLL > 5 µg/dL. Furthermore, none of the putative risk factors for elevated BLL was associated with BLL > 5 µg/dL. Intriguingly, in multivariate logistic regression analysis, a history of unexplained abdominal pain was associated with a four-fold risk of having BLL > 5 µg/dL. Lead levels from environmental specimens were also surprisingly within the recommended levels.

The overall mean ± SD BLL of 4.4 ± 0.75 µg/L was lower than the actionable limit of 5 µg/dL recommended by the Centres for Disease Control (CDC) [22, 23]. However, this mean BLL from our study is still five times higher than the 0.84 µg/dL reported in children living in the USA [10]. However, even low-level exposure to Pb is potentially deleterious in the long term. It is also noteworthy that the oldest participant in the current study was only 79 months old. Thus, given that lead is a cumulative toxicant, continued residence in the same community could eventually lead to even higher BLL especially if the children continue to be exposed to Pb-contaminated soil and dust. An overall 13.95% (*n* = 12) of the participants had BLL > 5 µg/dL, the recommended minimum cut-off point at which medical intervention is advised. Normally, when high BLL are reported, dietary changes, hand hygiene improvement, home improvement, and further investigations to identify other potential Pb sources are recommended for affected individuals. Affected children with BLL > 5 µg/dL should be re-tested 30–90 days after instituting Pb-lowering interventions [23].

Our results showed no significant difference between BLL by gender. This finding is consistent with a report by Nriagu et al. (1996) on Pb poisoning in children from KwaZulu Natal, South Africa [24]. That study reported no significant difference in BLL by gender in the age group 3 -5 years but found higher Pb levels in males in the age group 8 -10 years [24]. Our study had more males (*n* = 51) than females (*n* = 35) and was limited to participants within the age range of 6 months to 80 months. We found no difference in BLL by age possibly due to the lower ages of our participants. Other researchers however, reported a significant difference in BLL by age, with children aged 1 -4 years reported as having igher BLL compared to children aged below one year [25]. However, such differences were only observed when BLL exceeded 5 µg/dL. Perhaps the lack of concordance of our results with other reports from literature, could also be attributed to the low prevalence of BLL > 5.0 µg/dL in our study population and our sample size might also not have been large enough to detect underlying differences. More recently however, in concordance with our findings, a study by Zardast et al. (2020) also reported no significant difference in BLL by gender in children aged 1 -7 years [26].

The BLL of children who played with random objects was significantly higher than that of children who played with plastic toys (*p* = 0.04). This finding could suggest that children who do not have designated toys might use any accessible random items as toys. Some of objects could have been heavily contaminated with Pb. Designated toys are more likely than random objects to be kept clean thus reducing their Pb load. Furthermore, a study on the lead content of plastic toys reported that Chinese manufactured toys contained higher lead content compared to toys manufactured in Turkey indicating that toys might still be a major source of lead exposure [16]. In univariate logistic regression analysis, a history of unexplained abdominal pain was a marginal determinant of elevated BLL, but after adjusting for petrol exposure and toy preference, a history of abdominal pain became a significant determinant of elevated BLL, OR 4.39 (95%CI 1.07–17.9). Although unexplained abdominal pain could be a symptom of Pb exposure, this possibility is unlikely since abdominal symptoms are usually only observed at higher BLL (> 10 µg/dL). We were unable to inquire further into this observation but speculate that the abdominal pain could perhaps be attributed to non-Pb –related causes.

Parental/guardian occupational exposure is reported to be a risk factor for elevated BLL in children. In the current study, 15.1% (*n* = 13) of study participants’ parents/guardians worked in settings where they were exposed to high risk of Pb exposure (mainly metalworking and furniture making). There was however, no significant difference in mean BLL between participants from households with an occupationally exposed parent/guardian and those with parents/guardians that were not occupationally exposed. This finding could be attributed to personal hygiene practices by the occupationally exposed individuals or a safe work environment that minimised Pb exposure to the workers.

Pica, a common occurrence in young children, is associated with an increased risk of elevated BLL. Common causes of pica include mineral deficiency and hunger. Although we report the presence of appreciable amounts Pb in soil specimens and paint flakes, participant behaviour linked to pica such as soil eating, and finger sucking were not significantly associated with elevated BLL. Our findings are similar to those of a study conducted in Nairobi, Kenya, which reported a mean BLL of 5.9 µg/dL and in concordance with our findings, found no association between pica and BLL despite high soil Pb levels [27]. This finding could suggest alternative routes of Pb exposure besides ingestion inhalation and dermal absorption.

The banning of leaded fuels in Sub Saharan Africa (SSA) in 2006 led to a progressive decline in BLL in children residing in SSA urban areas. A case in point is the decline of BLL > 10 µg/dL in children living in Jos, Nigeria, from 70% in 2000 to 55% in 2005 and 44.7% in 2011 [28]. The BLL results in the present study ranging from 2.8–6.4 µg/dLwere in concordance with findings by Rollin et al. (2017) in a study conducted in South Africa [29]. Unfortunately, there is no prior data on BLL in Zimbabwean children with which present findings could be compared.

The Pb content in paint chips in the present study ranged from 9.64 – 169.24 mg/kg. The paint chips from the majority of apartments in our study site had higher Pb than that recommended by the US Consumer Product Safety Commission and the World Health Organisation (WHO) who recommend Pb levels < 90 mg/kg [30]. Lead levels in domestic water sources and environmental water puddles in the present study ranged from 0.3–3.3 µg/L which was within the WHO tolerable limits of < 10 µg/L [31]. Curiously, treated water from communal taps had higher Pb content compared to both borehole water and puddle water. This could be explained by the possibility of Pb leaching from the old plumbing laid down in the 1940s before the prohibition of Pb solder in linking water pipes. The water Pb level in the current study was however, lower than 40 µg/L reported by Gombiro et al. (2014) in commercial Zimbabwean mineral water in 2014 [32].

Soil Pb levels ranged from 3.34 – 177.5 mg/kg and were below the acceptable lower limit for total soil Pb of 250 mg/kg [33]. The soil Pb levels observed in the present study were surprisingly low given the putative potential Pb contaminants in the study area such as Pb laden paint flakes from old buildings, proximity to heavy industrial sites, and exposure to various cottage industries. However, the soil Pb extraction method utilised in this study only extracts bioavailable Pb which normally constitutes 70% of total Pb. It might therefore be possible that the total soil Pb level could be higher than the amount that was reported in the present study.

The Pb content of paint flake specimens in the present study ranged from 9.64-169 mg/kg, which was below the recommended 600 mg/kg but above the USA Consumer Product Safety Commission recommended paint flake Pb content of 90 mg/kg [30]. The paint flakes therefore remain a plausible low-level source of environmental Pb contamiantion since the paint flakes may contain bioaccessible Pb. The Pb content of both paint flakes and the soil specimens were however, in concordance with levels previously reported in other studies in other settings in SSA including Zimbabwe [27, 34].

### Limitations

A major strength of the study lay in the design and random nature of participant enrolment, which allows for the external validity of our findings. A major limitation of our study was however, the lack of a comparison group from a suburb that is located in an area that is less prone to environmental lead exposure. Furthermore, our sample size became constrained when we generated additional participant strata for statistical comparisons and some of the strata ended up with too few participants for effective comparison. Use of the USA Environmental Protection Agency Integrated Exposure Uptake Biokinetic Model for Lead in children could have provided further insights into the possible risk factors for elevated BLL. We were however unable to make use of this model since we had not collected some requisite data on exposure durations and lead levels in air and food samples. Finally, some potential confounders such as zinc and calcium deficiency were not objectively accounted for in the present study.

## Conclusions

Although the mean BLL reported in our study was lower than the actionable cut-off of 5 µg/dL, our findings are still a cause of concern and further evaluation to determine Pb exposure rates in vulnerable groups and communities is required. Despite our failure to demonstrate a causal association between putative risk factors and high BLL, our findings provide an initial evaluation, which if followed up, is useful to guide primary care physicians in making decisions regarding the management of paediatric patients.

## Data Availability

The datasets used and/or analysed during the current study available from the corresponding author on reasonable request.

## Abbreviations

Pb: Lead
BLL: Blood lead level
USA: United States of America
EDTA: Ethylenediaminetetraacetic acid
AAS: Atomic Absorption Spectrophotometry
ANOVA: Analysis of Variance
JREC: Joint Research Ethics Committee
SD: Standard Deviation
OR: Odds Ratio
CI: Confidence Interval
CDC: Centres for Disease Control
SSA: Sub Saharan Africa
WHO: World Health Organisation
